# Supplementary material for: Transport infrastructure severely impacts amphibian dispersal regardless of life stage
Source: Sci Rep. 2019 Jun 3;9:8214. doi: 10.1038/s41598-019-44706-1 (PMC6546755; doi:10.1038/s41598-019-44706-1)
Supplement: Supplementary file 1 — Supplementary material [file 41598_2019_44706_MOESM1_ESM.docx]

**SUPPLEMENTARY**

**Transport infrastructure severely impacts amphibian dispersal regardless of life stage**

Hugo Cayuela, Éric Bonnaire, Guillelme Astruc, Aurélien Besnard

Table S1. Description of the 37 states of the multievent CR model.

| State | Description |
| --- | --- |
| jSo | Juvenile, not captured at *t*, stayed in the same patch between *t*–1 and *t* |
| ojS+ | Juvenile, not captured at *t*–1, captured at *t*, stayed in the same patch between *t*–1 and *t* |
| +jS+ | Juvenile, captured at *t*–1 and *t*, stayed in the same patch between *t*–1 and *t* |
| jM1o | Juvenile, not captured at *t*, moved to another patch between *t*–1 and *t* located at a distance of 100–800 m from the source patch |
| jM1+ | Juvenile, not captured at *t*–1, captured at *t*, moved to another patch between *t*–1 and *t* located at a distance of 100– 800 m from the source patch |
| +jM1+ | Juvenile, captured at *t*–1 and *t*, moved to another patch between *t*–1 and *t* located at a distance of 100–800 m from the source patch |
| jM2o | Juvenile, not captured at *t*, moved to another patch between *t*–1 and *t* located at a distance of 800–1500 m from the source patch |
| jM2+ | Juvenile, not captured at *t*–1, captured at *t*, moved to another patch between *t*–1 and *t* located at a distance of 800– 1500 m from the source patch |
| +jM2+ | Juvenile, captured at *t*–1 and *t*, moved to another patch between *t*–1 and *t* located at a distance of 800–1500 m from the source patch |
| jM3o | Juvenile, not captured at *t*, moved to another patch between *t*–1 and *t* located at a distance of more than 1500 m from the source patch |
| jM3+ | Juvenile, not captured at *t*–1, captured at *t*, moved to another patch between *t*–1 and *t* located at a distance of more than 1500 m from the source patch |
| +jM3+ | Juvenile, captured at *t*–1 and *t*, moved to another patch between *t*–1 and *t* located at a distance of more than 1500 m from the source patch |
| sSo | Subadult, not captured at *t*, stayed in the same patch between *t*–1 and *t* |
| osS+ | Subadult, not captured at *t*–1, captured at *t*, stayed in the same patch between *t*–1 and *t* |
| +sS+ | Subadult, captured at *t*–1 and *t*, stayed in the same patch between *t*–1 and *t* |
| sM1o | Subadult, not captured at *t*, moved to another patch between *t*–1 and *t* located at a distance of 100–800 m from the source patch |
| sM1+ | Subadult, not captured at *t*–1, captured at *t*, moved to another patch between *t*–1 and *t* located at a distance of 100–800 m from the source patch |
| +sM1+ | Subadult, captured at *t*–1 and *t*, moved to another patch between *t*–1 and *t* located at a distance of 100–800 m from the source patch |
| sM2o | Subadult, not captured at *t*, moved to another patch between *t*–1 and *t* located at a distance of 800–1500 m from the source patch |
| sM2+ | Subadult, not captured at *t*–1, captured at *t*, moved to another patch between *t*–1 and *t* located at a distance of 800– 1500 m from the source patch |
| +sM2+ | Subadult, captured at *t*–1 and *t*, moved to another patch between *t*–1 and *t* located at a distance of 800–1500 m from the source patch |
| sM3o | Subadult, not captured at *t*, moved to another patch between *t*–1 and *t* located at a distance of more than 1500 m from the source patch |
| sM3+ | Subadult, not captured at *t*–1, captured at *t*, moved to another patch between *t*–1 and *t* located at a distance of more than 1500 m from the source patch |
| +sM3+ | Subadult, captured at *t*–1 and *t*, moved to another patch between *t*–1 and *t* located at a distance of more than 1500 m from the source patch |
| aSo | Adult, not captured at *t*, stayed in the same patch between *t*–1 and *t* |
| oaS+ | Adult, not captured at *t*–1, captured at *t*, stayed in the same patch between *t*–1 and *t* |
| +aS+ | Adult, captured at *t*–1 and *t*, stayed in the same patch between *t*–1 and *t* |
| aM1o | Adult, not captured at *t*, moved to another patch between *t*–1 and *t* located at a distance of 100–800 m from the source patch |
| aM1+ | Adult, not captured at *t*–1, captured at *t*, moved to another patch between *t*–1 and *t* located at a distance of 100–800 m from the source patch |
| +aM1+ | Adult, captured at *t*–1 and *t*, moved to another patch between *t*–1 and *t* located at a distance of 100–800 m from the source patch |
| aM2o | Adult, not captured at *t*, moved to another patch between *t*–1 and *t* located at a distance of 800–1500 m from the source patch |
| aM2+ | Adult, not captured at *t*–1, captured at *t*, moved to another patch between *t*–1 and *t* located at a distance of 800–1500 m from the source patch |
| +aM2+ | Adult, captured at *t*–1 and *t*, moved to another patch between *t*–1 and *t* located at a distance of 800–1500 m from the source patch |
| aM3o | Adult, not captured at *t*, moved to another patch between *t*–1 and *t* located at a distance of more than 1500 m from the source patch |
| aM3+ | Adult, not captured at *t*–1, captured at *t*, moved to another patch between *t*–1 and *t* located at a distance of more than 1500 m from the source patch |
| +aM3+ | Adult, captured at *t*–1 and *t*, moved to another patch between *t*–1 and *t* located at a distance of more than 1500 m from the source patch |
| D | Dead |

Table S2. Description of the 15 events of the multievent CR model

| Event | Description |
| --- | --- |
| 0 | Not captured at *t* |
| 1 | Juvenile, not captured at *t*–1 |
| 2 | Juvenile, captured in the same patch as at *t*–1 |
| 3 | Juvenile, captured in a different patch than at *t*–1 located at a distance of 100–800 m from the source patch |
| 4 | Juvenile, captured in a different patch than at *t*–1 located at a distance of 800–1500 m from the source patch |
| 5 | Juvenile, captured in a different patch than at *t*–1 located at a distance of more than 1500 m from the source patch |
| 6 | Subadult, not captured at *t*–1 |
| 7 | Subadult, captured in the same patch as at *t*–1 |
| 8 | Subadult, captured in a different patch than at *t*–1 located at a distance of 100–800 m from the source patch |
| 9 | Subadult, captured in a different patch than at *t*–1 located at a distance of 800–1500 m from the source patch |
| 10 | Subadult, captured in a different patch than at *t*–1 located at a distance of more than 1500 m from the source patch |
| 11 | Adult, not captured at *t*–1 |
| 12 | Adult, captured in the same patch as at *t*–1 |
| 13 | Adult, captured in a different patch than at *t*–1 located at a distance of 100–800 m from the source patch |
| 14 | Adult, captured in a different patch than at *t*–1 located at a distance of 800–1500 m from the source patch |
| 15 | Adult, captured in a different patch than at *t*–1 located at a distance of more than 1500 m from the source patch |

Table S3. Age-dependent dispersal distance. Model selection procedure to test the effect of AGE (three stages: juvenile, subadult and adult) and YEAR (from 2012 to 2016) on survival (ϕ), departure (ψ), arrival (α) and recapture (*p*) probabilities. r = model rank, k = number of parameters, Dev. = residual deviance, AICc = Akaike information criterion adjusted for small sample size, ΔAICc = difference of AICc points, w = AICc weight. Sample size = 8474 individuals.

| *r* | Model | k | Dev. | AICc | ΔAICc | w |
| --- | --- | --- | --- | --- | --- | --- |
| 1 | ϕ(AGE), ψ(AGE), α (AGE), *p*(AGE × YEAR) | 31 | 62,563.56 | 62,625.68 | 0.00 | 1.00 |
| 2 | ϕ(AGE), ψ(.), α (AGE), *p*(AGE × YEAR) | 29 | 62,569.70 | 62,637.90 | 12.22 | 0.00 |
| 3 | ϕ(AGE), ψ(AGE), α (AGE), *p*(YEAR) | 21 | 62,551.17 | 62,693.23 | 67. 55 | 0.00 |
| 4 | ϕ(AGE), ψ(.), α (AGE), *p*(YEAR) | 19 | 62,557.31 | 62,695.36 | 69.68 | 0.00 |
| 5 | ϕ(.), ψ(AGE), α (AGE), *p*(AGE × YEAR) | 29 | 62,641.55 | 62,699.90 | 74.22 | 0.00 |
| 6 | ϕ(.), ψ(.), α (AGE), *p*(AGE × YEAR) | 27 | 62,647.69 | 62,701.78 | 76.10 | 0.00 |
| 7 | ϕ(.), ψ(AGE), α (AGE), *p*(YEAR) | 19 | 62,764.92 | 62,802.96 | 177.28 | 0.00 |
| 8 | ϕ(.), ψ(.), α (AGE), *p*(YEAR) | 17 | 62,771.06 | 62,805.09 | 179.41 | 0.00 |
| 9 | ϕ(AGE), ψ(AGE), α (AGE), *p*(AGE) | 18 | 62,991.51 | 63,027.55 | 401.87 | 0.00 |
| 10 | ϕ(AGE), ψ(AGE), α (AGE), *p*(AGE) | 19 | 63,015.97 | 63,054.02 | 428.34 | 0.00 |
| 11 | ϕ(AGE), ψ(.), α (AGE), *p*(AGE) | 17 | 63,022.11 | 63,056.15 | 430.47 | 0.00 |
| 12 | ϕ(AGE), ψ(AGE), α (AGE), *p*(.) | 17 | 63,057.06 | 63,091.10 | 465.42 | 0.00 |
| 13 | ϕ(AGE), ψ(.), α (AGE), *p*(.) | 15 | 63,063.21 | 63,093.23 | 467.55 | 0.00 |
| 14 | ϕ(.), ψ(.), α (AGE), *p*(.) | 15 | 63,103.96 | 63,133.99 | 508.31 | 0.00 |
| 15 | ϕ(.), ψ(AGE), α (AGE), *p*(.) | 15 | 63,184.78 | 63,214.84 | 589.16 | 0.00 |
| 16 | ϕ(.), ψ(.), α (AGE), *p*(.) | 13 | 63,190.92 | 63,216.94 | 591.26 | 0.00 |

Table S4. Influence of gravel tracks on age-dependent dispersal. Model selection procedure to test the effect of AGE (three stages: juvenile, subadult and adult) and YEAR (from 2012 to 2016) on survival (ϕ), departure (ψ), arrival (α) and recapture (*p*) probabilities. Sample size = 8474 individuals.

| *r* | Model | k | Dev. | AICc | ΔAICc | w |
| --- | --- | --- | --- | --- | --- | --- |
| 1 | ϕ(AGE), ψ(AGE), α (AGE), *p*(AGE × YEAR) | 28 | 62,636.66 | 62,692.73 | 0.00 | 0.74 |
| 2 | ϕ(AGE), ψ(.), α (AGE), *p*(AGE × YEAR) | 26 | 62,642.77 | 62,694.86 | 12.22 | 0.26 |
| 3 | ϕ(AGE), ψ(AGE), α (AGE), *p*(YEAR) | 18 | 62,724.25 | 62,760.29 | 67. 55 | 0.00 |
| 4 | ϕ(AGE), ψ(.), α (AGE), *p*(YEAR) | 16 | 62,730.39 | 62,762.42 | 69.68 | 0.00 |
| 5 | ϕ(.), ψ(AGE), α (AGE), *p*(AGE × YEAR) | 26 | 62,714.63 | 62,766.71 | 74.22 | 0.00 |
| 6 | ϕ(.), ψ(.), α (AGE), *p*(AGE × YEAR) | 24 | 62,720.76 | 62,768.84 | 76.10 | 0.00 |
| 7 | ϕ(.), ψ(AGE), α (AGE), *p*(YEAR) | 16 | 62,837.98 | 62,870.02 | 177.28 | 0.00 |
| 8 | ϕ(.), ψ(.), α (AGE), *p*(YEAR) | 14 | 62,844.13 | 62,872.15 | 179.41 | 0.00 |
| 9 | ϕ(AGE), ψ(AGE), α (AGE), *p*(AGE) | 16 | 63,089.05 | 63,121.08 | 401.87 | 0.00 |
| 10 | ϕ(AGE), ψ(.), α (AGE), *p*(AGE) | 14 | 63,095.19 | 63,123.22 | 428.34 | 0.00 |
| 11 | ϕ(AGE), ψ(AGE), α (AGE), *p*(.) | 14 | 63,130.14 | 63,158.16 | 430.47 | 0.00 |
| 11 | ϕ(AGE), ψ(.), α (AGE), *p*(.) | 12 | 63,136.28 | 63,160.30 | 465.42 | 0.00 |
| 13 | ϕ(.), ψ(AGE), α (AGE), *p*(AGE) | 14 | 63,170.89 | 63,198.92 | 467.55 | 0.00 |
| 14 | ϕ(.), ψ(.), α (AGE), *p*(AGE) | 12 | 63,177.03 | 63,201.05 | 508.31 | 0.00 |
| 15 | ϕ(.), ψ(AGE), α (AGE), *p*(.) | 12 | 63,257.85 | 63,281.87 | 589.16 | 0.00 |
| 16 | ϕ(.), ψ(.), α (AGE), *p*(.) | 10 | 63,263.99 | 63,284.00 | 591.26 | 0.00 |

Table S5. Influence of paved roads on age-dependent dispersal. Model selection procedure to test the effect of AGE (three stages: juvenile, subadult and adult) and YEAR (from 2012 to 2016) on survival (ϕ), departure (ψ), arrival (α) and recapture (*p*) probabilities. Sample size = 8474 individuals.

| *r* | Model | k | Dev. | AICc | ΔAICc | w |
| --- | --- | --- | --- | --- | --- | --- |
| 1 | ϕ(AGE), ψ(AGE), α (AGE), *p*(AGE × YEAR) | 28 | 62,411.61 | 62,467.71 | 0.00 | 0.74 |
| 2 | ϕ(AGE), ψ(.), α (AGE), *p*(AGE × YEAR) | 26 | 62,417.75 | 62,469.83 | 12.22 | 0.26 |
| 3 | ϕ(AGE), ψ(AGE), α (AGE), *p*(YEAR) | 18 | 62,499.23 | 62,535.27 | 67. 55 | 0.00 |
| 4 | ϕ(AGE), ψ(.), α (AGE), *p*(YEAR) | 16 | 62,505.37 | 62,537.40 | 69.68 | 0.00 |
| 5 | ϕ(.), ψ(AGE), α (AGE), *p*(AGE × YEAR) | 26 | 62,489.61 | 62,541.69 | 74.22 | 0.00 |
| 6 | ϕ(.), ψ(.), α (AGE), *p*(AGE × YEAR) | 24 | 62,495.74 | 62,543.82 | 76.10 | 0.00 |
| 7 | ϕ(.), ψ(AGE), α (AGE), *p*(YEAR) | 16 | 62,612.97 | 62,645.00 | 177.28 | 0.00 |
| 8 | ϕ(.), ψ(.), α (AGE), *p*(YEAR) | 14 | 62,619.11 | 62,647.13 | 179.41 | 0.00 |
| 9 | ϕ(AGE), ψ(AGE), α (AGE), *p*(AGE) | 16 | 62,864.03 | 62,896.06 | 401.87 | 0.00 |
| 10 | ϕ(AGE), ψ(.), α (AGE), *p*(AGE) | 14 | 62,870.17 | 62,898.19 | 428.34 | 0.00 |
| 11 | ϕ(AGE), ψ(AGE), α (AGE), *p*(.) | 14 | 62,905.12 | 62,933.14 | 430.47 | 0.00 |
| 12 | ϕ(AGE), ψ(.), α (AGE), *p*(.) | 12 | 62,911.26 | 62,935.27 | 465.42 | 0.00 |
| 13 | ϕ(.), ψ(AGE), α (AGE), *p*(AGE) | 14 | 62,945.87 | 62,973.90 | 467.55 | 0.00 |
| 14 | ϕ(.), ψ(.), α (AGE), *p*(AGE) | 12 | 62,952.01 | 62,976.03 | 508.31 | 0.00 |
| 15 | ϕ(.), ψ(AGE), α (AGE), *p*(.) | 12 | 63,032.83 | 63,056.85 | 589.16 | 0.00 |
| 16 | ϕ(.), ψ(.), α (AGE), *p*(.) | 10 | 63,038.97 | 63,058.98 | 591.26 | 0.00 |

Table S6. Lagrange model to examine sex-dependent dispersal. Lagrange model parameters are survival (ϕ), dispersal (ψ), and recapture (*p*) probabilities. The sex was not included in the best-supported model [ϕ(.), ψ(.), p(sex)]. The intra-annual dispersal rate extracted from the model [ϕ(.), ψ(sex), p(sex)] was 0.04 (95% CI 0.03-0.06) in females and 0.05 (95% CI 0.04-0.07) in males. The intra-annual dispersal rate was 0.15 (95% CI 0.10-0.20) in females and 0.16 (95% CI 0.11-0.22) in males.

| Model | k | Dev. | AICc |
| --- | --- | --- | --- |
| ϕ(.), ψ(.), *p*(sex) | 5 | 10,412.97 | 10,422.97 |
| ϕ(.), ψ(sex), *p*(sex) | 6 | 10,412.35 | 10,424.36 |
| ϕ(.), ψ(.), *p*(.) | 4 | 10,417.32 | 10,425.33 |
| ϕ(.), ψ(sex), *p*(.) | 5 | 10,416.69 | 10,426.71 |
